# A novel mistranslating tRNA model in *Drosophila melanogaster* has diverse, sexually dimorphic effects

**DOI:** 10.1093/g3journal/jkac035

**Published:** 2022-02-10

**Authors:** Joshua R Isaacson, Matthew D Berg, Brendan Charles, Jessica Jagiello, Judit Villén, Christopher J Brandl, Amanda J Moehring

**Affiliations:** 1 Department of Biology, The University of Western Ontario, London, ON N6A 5B7, Canada; 2 Department of Biochemistry, The University of Western Ontario, London, ON N6A 5B7, Canada; 3 Department of Genome Sciences, University of Washington, Seattle, WA 98195, USA

**Keywords:** tRNA, mistranslation, *Drosophila melanogaster*, development, locomotion, proteostasis, deformity, sex-specific

## Abstract

Transfer RNAs (tRNAs) are the adaptor molecules required for reading the genetic code and producing proteins. Transfer RNA variants can lead to genome-wide mistranslation, the misincorporation of amino acids not specified by the standard genetic code into nascent proteins. While genome sequencing has identified putative mistranslating transfer RNA variants in human populations, little is known regarding how mistranslation affects multicellular organisms. Here, we create a multicellular model of mistranslation by integrating a serine transfer RNA variant that mistranslates serine for proline (${\text{tRNA}}_{\text{UGG},G26A}^{\text{Ser}}$) into the *Drosophila melanogaster* genome. We confirm mistranslation via mass spectrometry and find that ${\text{tRNA}}_{\text{UGG},G26A}^{\text{Ser}}$ misincorporates serine for proline at a frequency of ∼0.6% per codon. ${\text{tRNA}}_{\text{UGG},G26A}^{\text{Ser}}$ extends development time and decreases the number of flies that reach adulthood. While both sexes of adult flies containing ${\text{tRNA}}_{\text{UGG},G26A}^{\text{Ser}}$ present with morphological deformities and poor climbing performance, these effects are more pronounced in female flies and the impact on climbing performance is exacerbated by age. This model will enable studies into the synergistic effects of mistranslating transfer RNA variants and disease-causing alleles.

## Introduction

Mistranslation occurs when an amino acid that differs from what is specified by the standard genetic code is incorporated into nascent proteins. Mistranslation is implicated in various disease phenotypes. Editing-defective transfer RNA (tRNA) synthetases that induce mistranslation cause cardiac abnormalities and neurodegeneration in mice (Lee *et al.* 2006; Liu *et al.* 2014), and impaired locomotion, reduced lifespan, and neurodegeneration in flies (Lu *et al.* 2014). Ectopically expressed mistranslating tRNAs cause developmental deformities in zebrafish (Reverendo *et al.* 2014) and promote tumor growth in mouse cell lines (Santos *et al.* 2018). tRNA variants can cause mistranslation and are also directly linked to human disease, as mitochondrial tRNA variants cause MELAS and MERRF in humans (Goto *et al.* 1990; Shoffner *et al.* 1990). Despite the profound impact of mistranslation and the prevalence of cytoplasmic tRNA variants with the potential to mistranslate in humans (Berg *et al.* 2019a), the impact of these variants on the biology of multicellular organisms is not well described.

Mutations in tRNAs that cause mistranslation arise spontaneously and were identified initially in *Escherichia coli* as suppressors of nonsense and missense mutations (see e.g. Stadler and Yanofsky 1959; Gorini and Beckwith 1966; Goodman *et al.* 1968). Subsequently, mistranslating tRNAs have been identified as suppressors of deleterious phenotypes in other organisms (e.g. Goodman *et al.* 1977; Wills *et al.* 1983; Chiu and Morris 1997; El Meziane *et al.* 1998; Murakami *et al.* 2005). While no spontaneous tRNA variants have been detected through suppression screens in *Drosophila*, researchers have engineered amber suppressing tRNA^Tyr^ and tRNA^Leu^ variants, respectively, with a low level of amber stop codon suppression activity when integrated into the *Drosophila melanogaster* genome (Laski *et al.* 1989; Garza *et al.* 1990). In both cases sterility was noted.

Translation requires base pairing of the anticodon, the three nucleotides of the tRNA at positions 34, 35, and 36, with complementary codons in mRNA in the A site of the ribosome. Because the anticodon provides a direct link between the tRNA and its amino acid assignment, it is the main identity element for the aminoacylation (the attachment of an amino acid to the 3’ end of a tRNA) for most tRNAs (Giegé *et al.* 1998), with the exception of tRNA^Ser^, tRNA^Ala^, and tRNA^Leu^ (Hou and Schimmel 1988; Mcclain and Foss 1988; Normanly *et al.* 1992; Achsel and Gross 1993; Asahara *et al.* 1993; Breitschopf *et al.* 1995; Himeno *et al.* 1997). Changing the anticodon of the gene expressing a serine tRNA (tRNA^Ser^) does not affect aminoacylation but changes codon recognition (Garza *et al.* 1990; Geslain *et al.* 2010; Reverendo *et al.* 2014; Zimmerman *et al.* 2018; Berg *et al.* 2019b), resulting in mistranslation. In this study, we stably integrate the gene expressing a tRNA^Ser^ variant that mistranslates serine at proline codons into the *D.melanogaster* genome. Development time of flies containing the mistranslating tRNA was extended and fewer flies reached adulthood compared with wild-type flies. The tRNA variant increased the prevalence of morphological deformities in adult flies, with females being more severely affected than males. Mistranslation also impaired climbing performance. Cytosolic mistranslating tRNA variants thus impact multiple aspects of the biology of a multicellular organism and in a sex-specific manner.

## Materials and methods

### Fly husbandry and stocks

All fly stocks were obtained from the Bloomington *Drosophila* Stock Centre and maintained on standard Bloomington recipe food medium (Bloomington, Indiana) under a 14:10 light:dark cycle at 24°C and 70% relative humidity.

### Creating transgenic stocks

The gene encoding wild-type ${\text{tRNA}}_{\text{UGA}}^{\text{Ser}}$ (FlyBase ID: FBgn0050201) was amplified from genomic DNA using primers VK3400/VK3401 (primers are listed in Supplementary Table 1) and cloned into pCDF4, a kind gift from Dr Simon Bullock (Port *et al.* 2014), as a *Bgl*II/*Xba*I fragment to create pCB4222. The gene encoding tRNA^Ser^ with a proline UGG anticodon and G26A secondary mutation (${\text{tRNA}}_{\text{UGG},G26A}^{\text{Ser}}$) were made by 2-step mutagenic PCR with primers VK3400/VK3889 and VK3401/VK3890 and pCB4222 as a template. Products from the first round were amplified with primers VK3400/VK3401 and cloned as a *Bgl*II/*Xba*I fragment into pCDF4 to give pCB4250. Sequences of ${\text{tRNA}}_{\text{UGA}}^{\text{Ser}}$ and ${\text{tRNA}}_{\text{UGG},G26A}^{\text{Ser}}$ are found in Supplementary Fig. 1.

To create flies containing mistranslating tRNAs, a stock expressing phiC31 (ΦC31) integrase in the germ line and containing an *attP* site in the left arm of the second chromosome was used (stock no. 25709: *y^1^ v^1^ P{nos-phiC31\int.NLS}X; P{CaryP}attP40*). Plasmids were injected into *D. melanogaster* embryos (Isaacson 2018). Transgenic flies were identified by their wild-type eye color and balanced using stock no. 3703 (*w^1118^/Dp(1; Y)y^+^; CyO/nub^1^ b^1^ sna Sco lt^1^ stw^3^; MKRS/TM6B, Tb^1^*) and no. 76359 (*w^1118^; wg^Sp-1^/CyO, P{w^+mC^=2xTb^1^-RFP}CyO; MKRS/TM6B, Tb^1^*) to create final stocks of the following genotype: *w^1118^*; *P{CaryP}attP40[v^+^=tRNA]*/*CyO*, *P{w^+^mC=2xTb^1^-RFP}CyO*; *MKRS*/*TM6B*, *Tb^1^*. This was the genotype of the flies used in all experiments. After producing offspring, DNA was extracted from both parents of the final cross for PCR amplification using the primer set M13R and VK3400 for sequence confirmation.

### Complementation in *Saccharomyces cerevisiae*

The *Bgl*II/*Xba*I fragment of pCB422 encoding *Drosophila*${\text{tRNA}}_{\text{UGG},G26A}^{\text{Ser}}$ was cloned into the *Bam*HI/*Xba*I sites of the yeast–*E. coli* shuttle plasmid YEPlac181 (pCB4877, Gietz and Sugino 1988). pCB4877 and YEPlac181 were transformed into the yeast strain CY9013 [*MATα his3Δ1 leu2Δ0 lys2Δ0 met15Δ0 ura3Δ0 tti2Δ-met5Δ-mTn10luk* containing pRS313 (Sikorski and Hieter 1989) expressing *tti2-L187P* (Berg *et al.* 2017)] selecting for growth on minimal plates lacking leucine and histidine. Transformants were streaked onto yeast–peptone (YP) plates containing 2% glucose and 5% ethanol and grown at 30°C for 4 days.

### Mass spectrometry

Detailed mass spectrometry protocols are described in the Supplementary Appendix. Briefly, protein was extracted from 20 pupae per sample, reduced, alkylated, and digested into peptides following the R2-P1 method as described in Leutert *et al.* (2019). Peptides were analyzed on a hybrid quadrupole orbitrap mass spectrometer (Orbitrap Exploris 480; Thermo Fisher Scientific). MS/MS spectra were searched against the *D. melanogaster* protein sequence database (downloaded from Uniprot in 2016) using Comet (release 2015.01; Eng *et al.* 2013). Mistranslation frequency was calculated using the unique mistranslated peptides for which the nonmistranslated sibling peptide was also observed and defined as the counts of mistranslated peptides, where serine was inserted for proline, divided by the counts of all peptides containing proline, respectively, expressed as a percentage.

### Scoring deformities

Virgin, heterozygous flies were collected within ∼8 h of eclosion and scored for deformities in adult legs (limbs gnarled or missing segments), wings (blistered, absent, fluid-filled, or abnormal size), or abdomen (fused or incomplete tergites). Flies collected before wing expansion were excluded. Sex and type of deformity was recorded. Flies with multiple deformities had each recorded. Four hundred and thirty-three ${\text{tRNA}}_{\text{UGA}}^{\text{Ser}}$ flies (227 males and 216 females) and 656 ${\text{tRNA}}_{\text{UGG},G26A}^{\text{Ser}}$ flies (345 male and 311 female) were scored. All deformities were photographed through the lens of a stereomicroscope using a Samsung Galaxy S8 camera.

### Developmental assays

Approximately 250 flies of each genotype (*w^1118^*; *P{CaryP}attP40[v^+^=${\text{tRNA}}_{\text{UGA}}^{\text{Ser}}$]*/*CyO*, *P{w^+^mC = 2xTb^1^-RFP}CyO*; *MKRS*/*TM6B*, *Tb^1^* or *w^1118^*; *P{CaryP}attP40[v^+^=${\text{tRNA}}_{\text{UGG},G26A}^{\text{Ser}}$]*/*CyO*, *P{w^+^mC = 2xTb^1^-RFP}CyO*; *MKRS*/*TM6B*, *Tb^1^)* were placed into fly cages and allowed to lay eggs for 1 h. Seven replicates of 30 eggs from each plate were checked every 12 h to record progress through development. Sex, zygosity, and deformities of adults were recorded.

### Climbing assays

Virgin adult flies were sorted by sex and scored for deformities. Deformed flies or flies homozygous for the transgenic tRNA were discarded. Equal numbers were collected from each genotype during each collection period. Sixty flies in 11 vials from each genotype were transferred to new food the day before testing. The number of flies that climbed to a 5-cm line in 10 s was recorded. Flies were retested every 3 days until the flies were 51 days old. Each vial was tested 3 times.

### Statistical analyses

Statistical analyses were performed using R Studio 1.2.5001. Analyses used for comparisons were: *t*-test (frequency of proline-to-serine misincorporation between ${\text{tRNA}}_{\text{UGA}}^{\text{Ser}}$ and ${\text{tRNA}}_{\text{UGG},G26A}^{\text{Ser}}$); Wilcoxon rank-sum tests (developmental time data, corrected using Holm–Bonferroni’s method); Fisher’s exact tests (survival between developmental stages and proportion of deformities, corrected using Holm–Bonferroni’s method). A generalized linear model was constructed from the climbing assay data and performance was compared using *F*-tests corrected using Bonferroni’s method.

## Results

### A tRNA^Ser^ variant induces mistranslation in D. melanogaster

**Fig. 1.**
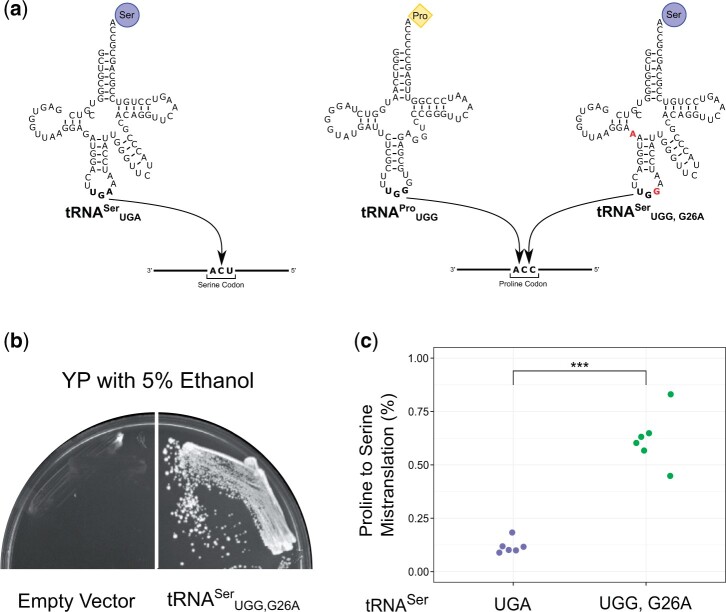
${\text{tRNA}}_{\text{UGG},G26A}^{\text{Ser}}$ induces mistranslation in *D. melanogaster*. a) Wild-type tRNA^Ser^ base pairs with serine codons, incorporating serine into the growing polypeptide. ${\text{tRNA}}_{\text{UGG},G26A}^{\text{Ser}}$ competes with ${\text{tRNA}}_{\text{UGG}}^{\text{Pro}}$ for CCA codons and inserts serine at proline codons. Red bases indicate mutation compared with the wild-type ${\text{tRNA}}_{\text{UGA}}^{\text{Ser}}$. b) *Drosophila melanogaster*${\text{tRNA}}_{\text{UGG},G26A}^{\text{Ser}}$ suppresses the ethanol sensitivity caused by *tti2-L187P* in *S. cerevisiae.* Plasmid encoding the vector alone (left) or the gene expressing ${\text{tRNA}}_{\text{UGG},G26A}^{\text{Ser}}$ (right) were transformed into CY9013 (*tti2-L187P*), streaked onto YP medium containing 5% ethanol and grown at 30°C for 4 days. c) Frequency of proline-to-serine mistranslation in ${\text{tRNA}}_{\text{UGA}}^{\text{Ser}}$ and ${\text{tRNA}}_{\text{UGG},G26A}^{\text{Ser}}$ pupae (*n* = 6 replicates of 20 pupae each). Genotypes were compared using a *t*-test. “***” *P* < 0.001.

To characterize mistranslation in a multicellular organism, we integrated genes encoding wild-type ${\text{tRNA}}_{\text{UGA}}^{\text{Ser}}$ as a control and a tRNA^Ser^ variant that mistranslates serine for proline (Fig. 1a) into the left arm of the second chromosome of the *D. melanogaster* genome. The tRNA^Ser^ variant has a proline UGG anticodon and G26A secondary mutation (${\text{tRNA}}_{\text{UGG},G26A}^{\text{Ser}}$). The alleles were balanced over a homolog that has serial inversions, preventing recombinant offspring, and transgene loss. tRNA insertions were validated with PCR using primers specific to the inserted plasmid and confirmed by sequencing. The secondary G26A mutation was included in the mistranslating tRNA to dampen tRNA function as we have previously found a tRNA^Ser^ variant with a proline anticodon causes lethal levels of mistranslation when expressed in yeast (Berg *et al.* 2017).

Adults homozygous for ${\text{tRNA}}_{\text{UGA}}^{\text{Ser}}$ or ${\text{tRNA}}_{\text{UGG},G26A}^{\text{Ser}}$ can be produced. However, we were unable to propagate the strain homozygous for ${\text{tRNA}}_{\text{UGG},\text{G26A}}^{\text{Ser}}$ because crosses between male and female ${\text{tRNA}}_{\text{UGG},G26A}^{\text{Ser}}$ homozygotes produce no viable offspring. As such, we used heterozygous flies for our experiments with adults. Studying heterozygous flies may be more biologically relevant as mistranslating tRNAs present in populations are likely to arise as single alleles. We determined zygosity by balancing the tRNAs over a *CyO* homolog containing Tubby-linked RFP and *miniwhite* (Pina and Pignoni 2012). Heterozygous larvae and pupae are identified by the presence of RFP and heterozygous adults by their curly wings and nonwhite eyes.

As an initial test of mistranslation by *Drosophila*${\text{tRNA}}_{\text{UGG},G26A}^{\text{Ser}}$, we determined if the tRNA rescues growth of a *Saccharomyces cerevisiae* strain containing *tti2-L187P* (CY9013). The *tti2-L187P* allele contains a missense mutation converting a CUA codon for leucine to CCA for proline and results in the slow growth of yeast in medium containing 5% ethanol (Hoffman *et al.* 2017). Mistranslation of proline to serine rescues the growth of yeast cells in ethanol medium (Berg *et al.* 2017). The gene encoding *Drosophila*${\text{tRNA}}_{\text{UGG},G26A}^{\text{Ser}}$ was transformed into a yeast strain that contains *tti2-L187P* as the sole copy of *TTI2*. Cells were transformed with plasmid expressing *Drosophila*${\text{tRNA}}_{\text{UGG},G26A}^{\text{Ser}}$ or vector alone. As shown in Fig. 1b, *Drosophila*${\text{tRNA}}_{\text{UGG},G26A}^{\text{Ser}}$ enabled growth of the strain on medium containing 5% ethanol indicative of mistranslation by *Drosophila*${\text{tRNA}}_{\text{UGG},G26A}^{\text{Ser}}$.

We then analyzed the proteome of *D. melanogaster* pupae by mass spectrometry to determine the mistranslation frequency (Fig. 1c; Supplementary File 2). Pupae were used because of the extensive cellular remodeling and corresponding rapid changes in protein synthesis that occur during this stage (Mitchell *et al.* 1977; Mitchell and Petersen 1981), and the potential of mistranslation during this stage to influence adult traits such as anatomy or neuronal function. The frequency of proline to serine mistranslation, calculated as the ratio of peptides containing the mistranslated serine residue to peptides containing the cognate proline residue, was ∼0.6% in flies expressing ${\text{tRNA}}_{\text{UGG},G26A}^{\text{Ser}}$. In the control strain, the frequency of proline to serine substitutions was 0.1%.

### ${\text{tRNA}}_{\text{UGG},G26A}^{\text{Ser}}$ adversely affects D. melanogaster development

**Fig. 2.**
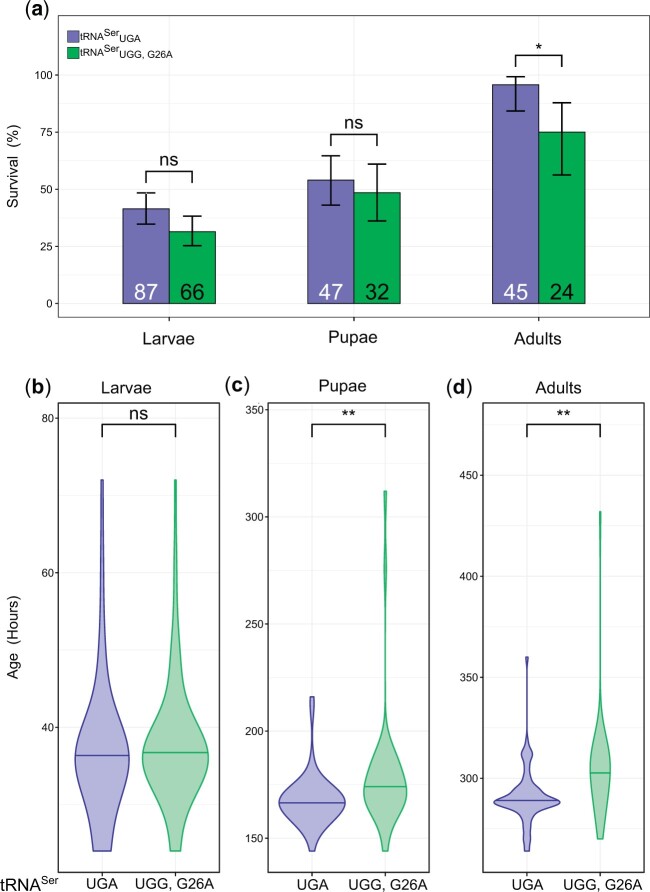
A mistranslating tRNA variant impacts development of *D. melanogaster*. a) Percentage of the 210 embryos containing ${\text{tRNA}}_{\text{UGA}}^{\text{Ser}}$ or ${\text{tRNA}}_{\text{UGG},G26A}^{\text{Ser}}$ that reached larval, pupal, and adult stages out of survivors from the previous stage. Survival was compared using Fisher’s exact test corrected using Holm–Bonferroni’s method. Error bars represent the 95% confidence interval of the proportion. Values within bars represent the number of flies that reached that developmental stage. b) Violin plot depicting the distribution of times for ${\text{tRNA}}_{\text{UGA}}^{\text{Ser}}$ and ${\text{tRNA}}_{\text{UGG},G26A}^{\text{Ser}}$ embryos to hatch into larva. The horizontal line within the plot represents the median of the distribution. Genotypes were compared using Wilcoxon’s rank-sum tests corrected using Holm–Bonferroni’s method. c) Total time until pupation. d) Total time until eclosion. “ns” *P* ≥ 0.05; “*” *P* < 0.05; “**” *P* < 0.01; “***” *P* < 0.001.

To determine if ${\text{tRNA}}_{\text{UGG},G26A}^{\text{Ser}}$ affects fly development, we collected 210 wild-type ${\text{tRNA}}_{\text{UGA}}^{\text{Ser}}$ and ${\text{tRNA}}_{\text{UGG},G26A}^{\text{Ser}}$ 1-h old embryos, comparing survival at 12-h intervals through each developmental stage (Fig. 2a) and time to reach each stage (Fig. 2, b–d): embryos to larvae, larvae to pupae, and pupae to adults. Since the RFP marker used to determine tRNA zygosity is not expressed during early embryonic stages, both homozygotes and heterozygotes were pooled in this assay. While there were fewer female and homozygotic ${\text{tRNA}}_{\text{UGG},G26A}^{\text{Ser}}$ flies compared with ${\text{tRNA}}_{\text{UGA}}^{\text{Ser}}$ flies, neither the male bias nor heterozygote bias reached statistical significance (Supplementary File 2). Figure 2a shows the percentage of individuals who reached a developmental stage relative to those who reached the previous stage (e.g. how many larvae managed to pupate). Of the 210 ${\text{tRNA}}_{\text{UGA}}^{\text{Ser}}$ embryos collected, 87 hatched into larvae, 47 larvae pupated, and 45 of those pupae reached adulthood. Survival of ${\text{tRNA}}_{\text{UGA}}^{\text{Ser}}$ was low due to the presence of three balancers (*CyO*, *MKRS*, and *TM6B*) in heterozygous flies and two (*MKRS* and *TM6B*) in homozygous flies. ${\text{tRNA}}_{\text{UGG},G26A}^{\text{Ser}}$ resulted in reduced viability at each stage as only 66 out of 210 embryos containing ${\text{tRNA}}_{\text{UGG},G26A}^{\text{Ser}}$ hatched, 32 larvae pupated, and 24 pupae reached adulthood. However, the difference between the proportion of ${\text{tRNA}}_{\text{UGA}}^{\text{Ser}}$ and ${\text{tRNA}}_{\text{UGG},G26A}^{\text{Ser}}$ embryos that hatched (41.4% vs 31.4%, *P* = 0.08, Fig. 2a) and the proportion of larvae that pupated (54.0% vs 48.5%, *P* = 0.51) was not statistically significant (Fisher’s exact test corrected using Bonferroni–Holm’s method). In contrast, the proportion of ${\text{tRNA}}_{\text{UGA}}^{\text{Ser}}$ pupae that reached adulthood was significantly higher than ${\text{tRNA}}_{\text{UGG},G26A}^{\text{Ser}}$ (95.7% vs 75.0%, *P* = 0.012). This indicates that flies are particularly susceptible to lethal effects of mistranslating tRNA variants during pupation.

Eggs expressing ${\text{tRNA}}_{\text{UGG},G26A}^{\text{Ser}}$ had similar hatching times as eggs expressing wild-type ${\text{tRNA}}_{\text{UGA}}^{\text{Ser}}$ (*P* = 0.24, Wilcoxon rank-sum test corrected using Holm–Bonferroni’s method, Fig. 2b). However, larvae expressing ${\text{tRNA}}_{\text{UGG},G26A}^{\text{Ser}}$ pupated significantly slower than the wild-type (*P* = 0.004, Fig. 2c). This trend continued into adulthood, as ${\text{tRNA}}_{\text{UGG},G26A}^{\text{Ser}}$ flies eclosed significantly later than control ${\text{tRNA}}_{\text{UGA}}^{\text{Ser}}$ flies (*P* = 0.002, Fig. 2d). Median development time of ${\text{tRNA}}_{\text{UGA}}^{\text{Ser}}$ flies was 288 h whereas median development time of ${\text{tRNA}}_{\text{UGG},G26A}^{\text{Ser}}$ flies was 303 h. Some extremely late pupation and eclosion events were observed in the mistranslating ${\text{tRNA}}_{\text{UGG},G26A}^{\text{Ser}}$ line and were a potential concern as they could have biased the statistical comparisons (Fig. 2, c and d; Supplementary File 2). However, ${\text{tRNA}}_{\text{UGG},G26A}^{\text{Ser}}$ flies still pupated and eclosed significantly later than ${\text{tRNA}}_{\text{UGA}}^{\text{Ser}}$ flies even after removing these values (*P* = 0.007 and *P* = 0.002, respectively, Supplementary Fig. 2). These results show that flies containing this mistranslating tRNA variant show extended development time and increased developmental lethality.

**Fig. 3.**
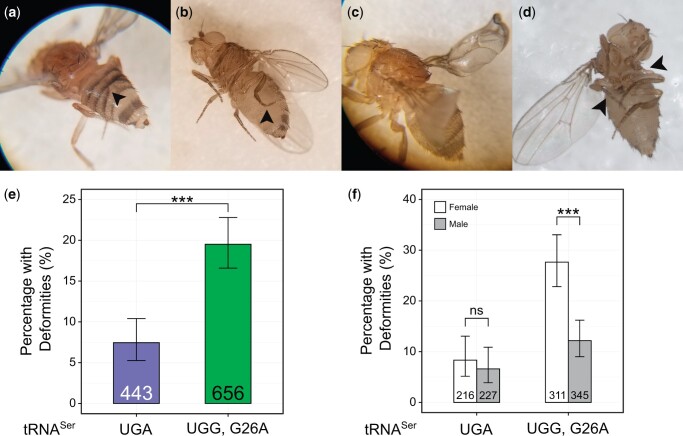
The ${\text{tRNA}}_{\text{UGG},G26A}^{\text{Ser}}$ variant causes morphological deformities in adults in a sex-specific manner. a) Examples of flies with misfused tergites; b) gnarled hindlegs; c) wing blisters; and d) missing wings/legs, as indicated by arrowheads. e) Percentage of ${\text{tRNA}}_{\text{UGA}}^{\text{Ser}}$ or ${\text{tRNA}}_{\text{UGG},G26A}^{\text{Ser}}$ flies that eclosed with at least 1 deformity. Groups were compared using Fisher’s exact test and corrected using Holm–Bonferroni’s method. Bar height represents the percentage of flies of a genotype that had at least 1 deformity. Error bars represent the 95% confidence interval. Values within bars describe the number of flies examined for deformities. f) Same data as (e) but separated by sex. “ns” *P* ≥ 0.05; “*” *P* < 0.05; “**” *P* < 0.01; “***” *P* < 0.001.

Mutations in genes vital to proteostasis or translation fidelity cause morphological defects (Rutherford and Lindquist 1998; Cui and DiMario 2007; Reverendo *et al.* 2014). We observed that flies containing one copy of the exogenous ${\text{tRNA}}_{\text{UGG},G26A}^{\text{Ser}}$ had deformities including gnarled or blistered legs, notched wings, and misfused tergites (Fig. 3, a–d). Other abnormalities (e.g. haltere aberrations or rough eyes) were rarely observed, so only the more common leg, wing, and tergite deformities were scored. To determine if the frequency of deformities was greater than the control, we calculated the proportion of flies that eclosed with at least one deformity. These flies were collected separately from the development assay described above. From a total of 433 ${\text{tRNA}}_{\text{UGA}}^{\text{Ser}}$ flies (227 males and 216 females) and 656 ${\text{tRNA}}_{\text{UGG},G26A}^{\text{Ser}}$ flies (345 males and 311 females) we identified proportionally more deformities in flies containing ${\text{tRNA}}_{\text{UGG},G26A}^{\text{Ser}}$ than ${\text{tRNA}}_{\text{UGA}}^{\text{Ser}}$ (Fisher’s exact test corrected using Holm–Bonferroni’s method, *P* < 0.001, Fig. 3e). In addition, female flies containing ${\text{tRNA}}_{\text{UGG},G26A}^{\text{Ser}}$ had more deformities than males (*P* < 0.001, Fig. 3f). Interestingly, flies containing ${\text{tRNA}}_{\text{UGG},G26A}^{\text{Ser}}$ presented with disproportionately more tergite deformities than flies with the wild-type ${\text{tRNA}}_{\text{UGA}}^{\text{Ser}}$ [chi-square test with post hoc analysis using the method outlined in (Shan and Gerstenberger 2017), *P* = 0.03, Supplementary File 3], indicating that this mistranslating tRNA^Ser^ variant is particularly deleterious to fly abdominal development. These results suggest that mistranslating tRNA variants can disrupt fly development and that female flies are more sensitive to their effects.

### ${\text{tRNA}}_{\text{UGG},G26A}^{\text{Ser}}$ impacts fly motility

**Fig. 4.**
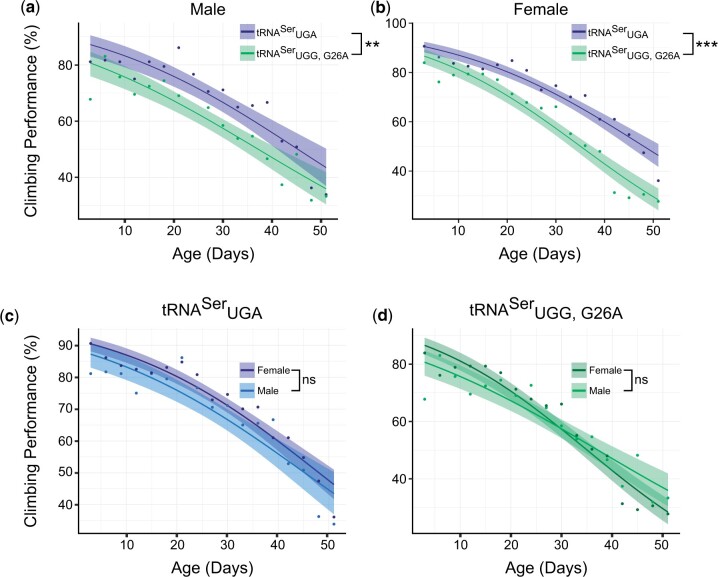
Fly locomotion is impacted by a mistranslating tRNA^Ser^ variant. Each point represents the percentage of flies (out of 60 individuals from 11 vials) that managed to climb 5 cm in 10 s averaged over 3 trials. Generalized linear models were constructed from the performance data and *F*-tests were performed on the models. *P*-values were corrected using Bonferroni’s method. Shaded region represents the 95% confidence intervals for the fitted performance curves. a) Climbing performance of male flies containing ${\text{tRNA}}_{\text{UGA}}^{\text{Ser}}$ or ${\text{tRNA}}_{\text{UGG},G26A}^{\text{Ser}}$. b) Climbing performance of female flies containing ${\text{tRNA}}_{\text{UGA}}^{\text{Ser}}$ or ${\text{tRNA}}_{\text{UGG},G26A}^{\text{Ser}}$. c) Climbing performance of male and female flies containing ${\text{tRNA}}_{\text{UGA}}^{\text{Ser}}$ or d) ${\text{tRNA}}_{\text{UGG},G26A}^{\text{Ser}}$. “ns” *P* ≥ 0.05; “*” *P* < 0.05; “**” *P* < 0.01; “***” *P* < 0.001.

Negative geotaxis assays are often used as an initial test of neurodegeneration in flies (e.g. 38–40); therefore, we determined if ${\text{tRNA}}_{\text{UGG},G26A}^{\text{Ser}}$ impaired climbing performance. Sixty virgin, heterozygous flies of the four genotypes (${\text{tRNA}}_{\text{UGA}}^{\text{Ser}}$ males and females, and ${\text{tRNA}}_{\text{UGG},G26A}^{\text{Ser}}$ males and females) were collected and tested using a climbing assay every three days; flies with deformities were not used in this experiment. Climbing performance of all genotypes decreased with age (*F*-tests performed on generalized linear models corrected using Bonferroni’s method, Supplementary File 2). For both males and females, climbing performance of ${\text{tRNA}}_{\text{UGG},G26A}^{\text{Ser}}$ flies was significantly worse than wild-type ${\text{tRNA}}_{\text{UGA}}^{\text{Ser}}$ flies (male: *P* = 0.001, female: *P* < 0.001, Fig. 4, a and b). Climbing performance was not significantly different when comparing males to females in either the control ${\text{tRNA}}_{\text{UGA}}^{\text{Ser}}$ (*P* = 0.08) or mistranslating ${\text{tRNA}}_{\text{UGG},G26A}^{\text{Ser}}$ flies (p → 1, Fig. 4, c and d). The climbing ability of male and female flies containing the wild-type ${\text{tRNA}}_{\text{UGA}}^{\text{Ser}}$ declined at similar rates, as evidenced by the parallel performance curves (*P* = 0.97, Fig. 4c). However, the climbing performance curve of female flies containing ${\text{tRNA}}_{\text{UGG},G26A}^{\text{Ser}}$ intersected the male curve, indicating a significant interaction effect between age and sex (*P* = 0.038, Fig. 4d; Supplementary File 3). Therefore, while overall climbing performance did not differ between ${\text{tRNA}}_{\text{UGG},G26A}^{\text{Ser}}$ males and females, rate of performance decline was faster for ${\text{tRNA}}_{\text{UGG},G26A}^{\text{Ser}}$ females. These data indicate that the mistranslating tRNA^Ser^ variant negatively affects locomotion and has an accelerated impact on female ability to climb as they age.

## Discussion

### A fly model of mistranslation

We have created a *D.melanogaster* model containing a genomically integrated cytosolic tRNA that mistranslates serine for proline. The mistranslating fly model allows for studies into sex-specific or tissue-specific effects of mistranslating tRNA variants and the effect of tRNA variants on development and disease. Our method of transgene integration controlled for positional effects by inserting either wild-type or mistranslating ${\text{tRNA}}_{\text{UGG},G26A}^{\text{Ser}}$ into the same locus on chromosome 2L. The fly lines containing ${\text{tRNA}}_{\text{UGG},G26A}^{\text{Ser}}$ have not lost the transgene for over two years, indicating that mistranslating tRNA variants can be stably maintained in the genome. We observed a proline-to-serine misincorporation rate of ∼0.6% in the pupae for the genomically integrated ${\text{tRNA}}_{\text{UGG},G26A}^{\text{Ser}}$ gene. This level of mistranslation was sufficient to cause deleterious phenotypes affecting diverse aspects of fly physiology.

### A mistranslating tRNA^Ser^ variant has diverse and sex-specific effects on flies

The mistranslating ${\text{tRNA}}_{\text{UGG},G26A}^{\text{Ser}}$ affects fly physiology consistent with organism-wide loss of proteostasis. Our findings resemble other studies of proteostasis loss in flies. Impaired heat shock response exacerbates neurodegeneration and increases development time (Warrick *et al.* 1999; Gong and Golic 2006), and many of the wing, leg, and tergite deformities observed for heterozygous *Heat shock protein 83* (*Hsp83*) mutants look similar to those observed in this study (Rutherford and Lindquist 1998). Developmental and neurodegenerative phenotypes including locomotive defects as measured in a climbing assay were likewise observed in flies containing a misacylation-prone PheRS (Lu *et al.* 2014). It is interesting to note that reduced levels of translation lead to similar deformities as found in mistranslating flies. RNAi knockdown of *Nopp140*, a gene involved in ribosome assembly, causes flies to present with gnarled legs, missing wings, and misfused tergites (Cui and DiMario 2007). *Minute* genes describe a collection of >50 genes required for protein synthesis. Their mutation results in shorter, thinner bristles, delayed development, smaller body size, and anatomical deformities when mutated (Schultz 1929; Marygold *et al.* 2007), again similar to the developmental and anatomical aberrations seen in flies containing the mistranslating tRNA^Ser^ variant. Though reduced translation and mistranslation are different processes, the similar phenotypes produced demonstrate that development is highly dependent on accurate and efficient translation.

The increased impact of the mistranslating tRNA on female flies was striking. *Drosophilamelanogaster* males and females have highly different physiology and experience different developmental challenges. Adult females are larger than males, develop faster, invest more resources into reproduction, and tend to live longer than males (Bonnier 1926; Bakker 1959; Sørensen *et al.* 2007; Ziehm *et al.* 2013). Males and females also display dimorphic responses to proteotoxic stress. Fredriksson *et al.* (2012) examined protein carbonylation in female somatic and germ line cells at different ages to determine how aging affects protein quality control of somatic and reproductive tissues (Fredriksson *et al.* 2012). They found that as females age, there are fewer carbonylated proteins and reduced protein aggregation (both indicators of proteostasis loss) in eggs compared with the soma. Their work shows that females prioritize maintaining proteostasis of their eggs over their somatic cells, even while unmated. This trade-off could exacerbate the stress of mistranslating tRNAs in females, particularly as they experience aging-induced loss of proteostasis, and could contribute to the faster decline of climbing performance observed in female ${\text{tRNA}}_{\text{UGG},G26A}^{\text{Ser}}$ flies compared with males. Many stress–response pathways affect males and females differently. For example, induction of the heat shock response increases male lifespan whereas female lifespan is unaffected (Sørensen *et al.* 2007, reviewed in Tower 2011). Dietary restriction shows the opposite trend, as it increases female lifespan more than male (Nakagawa *et al.* 2012; Regan *et al.* 2016; Garratt 2020). Experiments testing the effects of mistranslating tRNAs on male and female fly longevity are ongoing. It is also possible that expression of the mistranslating tRNA differs between males and females or that the mistranslating tRNA has alternative functions (e.g. tRNA-derived fragments) that differ between males and females.

### Implications for human disease

Our work suggests that mistranslating tRNA variants have the potential to influence multiple aspects of human physiology. From a development perspective, the alteration in progression through life stages and increased number of deformities suggest that the proteotoxic stress resulting from mistranslating tRNA variants may contribute to congenital or developmental anomalies. Flies expressing ${\text{tRNA}}_{\text{UGG},G26A}^{\text{Ser}}$ have a pattern of locomotion defects similar to those seen for flies expressing alleles associated with neurodegeneration (Feany and Bender 2000; Song *et al.* 2017; Aggarwal *et al.* 2019). Interestingly, the mistranslating fly model further resembles human neuropathies in that climbing performance declined faster in female compared with male flies, just as some neurodegenerative disorders, such as Alzheimer’s and Huntington’s disease, are more common or severe in women compared with men (Viña and Lloret 2010; Zielonka *et al.* 2013).

Given the prevalence of putative mistranslating tRNAs in the human population (Berg *et al.* 2019a) and the potential for mistranslation to disrupt proteostasis, we hypothesize that mistranslating tRNAs can exacerbate diseases characterized by a loss of proteostasis (see also Reverendo *et al*. 2014), and our results here indicate that these effects may differ in magnitude between sexes. Our previous studies in yeast have shown negative genetic interactions between mistranslation and mutations in genes involved in protein quality control and other pathways that could contribute to disease (Hoffman *et al.* 2017; Berg *et al.* 2020, 2021). Our *D. melanogaster* model of mistranslation allows for the expansion of these studies into the investigation of mutant tRNA contribution to disease and development.

## Data availability

Fly lines and plasmids are available upon request. The authors affirm that all data necessary for confirming the conclusions of the article are present within the article, figures, and supplementary material. Supplementary File 1 contains an extended methods section and supplementary figures and tables. Supplementary File 2 contains all raw data. Supplementary File 3 contains R code used to analyze mass spectrometry, developmental, deformity, and climbing assay data. The mass spectrometry proteomics data have been deposited to the ProteomeXchange Consortium via the PRIDE partner repository (Perez-Riverol *et al.* 2019) with the dataset identifier PXD028498.

Supplementary material is available at *G3* online.

## Supplementary Material

jkac035_File_S1

jkac035_File_S2

jkac035_File_S3
