# Supplementary material for: A novel mistranslating tRNA model in Drosophila melanogaster has diverse, sexually dimorphic effects
Source: G3 (Bethesda). 2022 Feb 10;12(5):jkac035. doi: 10.1093/g3journal/jkac035 (PMC9073681; doi:10.1093/g3journal/jkac035)
Supplement: jkac035_File_S1 [file jkac035_file_s1.docx]

**Supplemental Material**

**Extended Mass Spectrometry methods**

Liquid chromatography tandem mass spectrometry was performed on strains expressing mistranslating tRNA variants to identify mistranslation. Six replicates of twenty pupae were collected from each genotype and lysed in 8 M urea, 50 mM Tris, 75 mM NaCl, pH 8.2 by grinding with a pestle and with glass beads at 4°C. Protein was reduced with 5 mM dithiothreitol for 30 minutes at 55°C and alkylated with 15 mM iodoacetamine for 30 minutes at room temperature. Robotic purification and digestion of proteins into peptides were performed on the KingFisher^TM^ Flex using LysC and the R2-P1 method as described in Leutert *et al.* (2019). Peptides were analyzed on a hybrid quadrupole orbitrap mass spectrometer (Orbitrap Exploris 480; Thermo Fisher Scientific) equipped with an Easy1200 nanoLC system (Thermo Fisher Scientific). Peptide samples were resuspended in 4% acetonitrile, 3% formic acid and loaded onto a 100 μm ID × 3 cm precolumn packed with Reprosil C18 3 μm beads (Dr. Maisch GmbH) and separated by reverse-phase chromatography on a 100 μm ID × 30 cm analytical column packed with Reprosil C18 1.9 μm beads (Dr. Maisch GmbH) housed into a column heater set at 50°C.

Peptides were separated using a gradient of 5-30% acetonitrile in 0.125% formic acid at 400 nL/min over 95 min and online analyzed by tandem mass spectrometry with a total 120 minute acquisition time. The mass spectrometer was operated in data-dependent acquisition mode with a defined cycle time of 3 seconds. For each cycle one full mass spectrometry (MS) scan was acquired from 350 to 1200 m/z at 120,000 resolution with a fill target of 3E6 ions and automated calculation of injection time. The most abundant ions from the full MS scan were selected for fragmentation using 2 m/z precursor isolation window and beam-type collisional-activation dissociation (HCD) with 30% normalized collision energy. MS/MS spectra were acquired at 15,000 resolution by setting the AGC target to standard and injection time to automated mode. Fragmented precursors were dynamically excluded from selection for 60 seconds.

MS/MS spectra were searched against the *D. melanogaster* protein sequence database (downloaded from Uniprot in 2016) using Comet (release 2015.01; Eng *et al.* 2013). The precursor mass tolerance was set to 50 ppm. Constant modification of cysteine carbamidomethylation (57.0215 Da) and variable modification of methionine oxidation (15.9949 Da) and proline to serine (-10.0207 Da) were used for all searches. A maximum of two of each variable modification were allowed per peptide. Search results were filtered to a 1% false discovery rate at the peptide spectrum match level using Percolator (Käll *et al.* 2007). The mistranslation frequency was calculated using the unique mistranslated peptides for which the non-mistranslated sibling peptide was also observed. The frequency is defined as the counts of mistranslated peptides, where serine was inserted for proline, divided by the counts of all peptides containing proline, respectively, and expressed as a percentage.

**Table S1.** Primers used in this study.

| Primer name | Sequence |
| --- | --- |
| VK3400 | GGGAGATCTGGTATGAAGCATAGATTTCAGC |
| VK3401 | AAATCTAGACCCGCACGGGAAATTCCTAGG |
| VK3889 | AATGGACTTGGAATCCATTGGGTTCTACCCG |
| VK3890 | CCAATGGATTCCAAGTCCATTTCCTTAACCACTC |
| M13R | CAGGAAACAGCTATGACCATG |


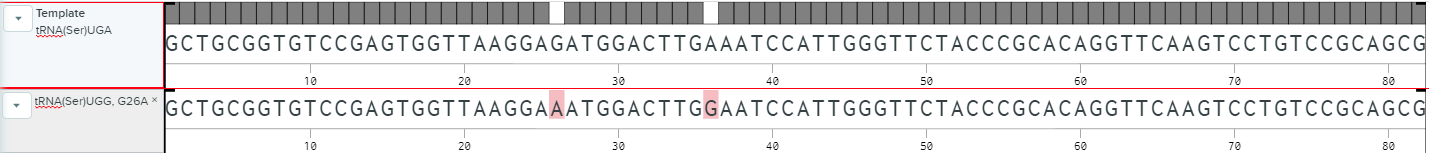
**Figure S1.** Sequence alignment of wild type tRNA^Ser^_UGA_ and tRNA^Ser^_UGG, G26A_. The sequence of the wild type tRNA^Ser^_UGA_ gene (FlyBase ID: FBgn0050201, top) compared to the engineered tRNA^Ser^_UGG, G26A_ used in this study (bottom). Highlighted bases represent differences between tRNA^Ser^_UGA_ and tRNA^Ser^_UGG, G26A_. Sequences were aligned using Benchling.

**
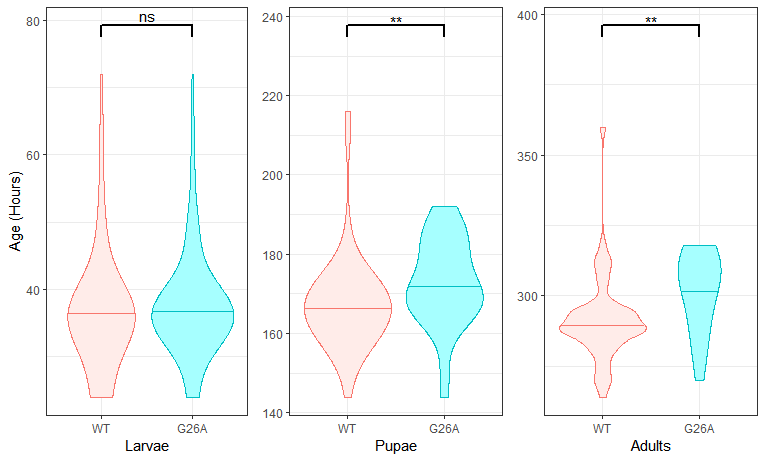
**

**Figure S2.** Violin plot depicting the distribution of times for tRNA^Ser^_UGA_ and tRNA^SerUGG, G26A^ embryos to become larvae (left), pupae (middle), or adults (right) excluding very late tRNA^Ser^_UGG, G26A_ pupation and eclosion events. “WT” refers to tRNA^Ser^_UGA_ and “G26A” refers to tRNA^Ser^_UGG, G26A_. The horizontal line within the plot represents the median of the distribution. Genotypes were compared using Wilcoxon rank-sum tests corrected using Holm-Bonferroni’s method. “ns” p ≥ 0.05, “*” p < 0.05, “**” p < 0.01, “***” p < 0.001.
